# Supplementary material for: QSAR Studies, Synthesis, and Biological Evaluation of New Pyrimido-Isoquinolin-Quinone Derivatives against Methicillin-Resistant Staphylococcus aureus
Source: Pharmaceuticals (Basel). 2023 Nov 17;16(11):1621. doi: 10.3390/ph16111621 (PMC10675065; doi:10.3390/ph16111621)
Supplement: Supplementary file 1 [file pharmaceuticals-16-01621-s001.zip › pharmaceuticals-2712095-supplementary.pdf]

Supplementary Material

QSAR Studies, Synthesis, and Biological Evaluation of New Pyrimido-Isoquinolin-Quinone Derivatives against Methicillin-Resistant *Staphylococcus aureus*

Juan Andrades-Lagos [1,2] , Javier Campanini-Salinas [2,3,*] , Gianfranco Sabadini [4,5], Victor Andrade [6,7,8], Jaime Mella [4,5,*] and David Vásquez-Velásquez [2,*]

Figure S1. The Chemical structure of the compounds used to construct the QSAR models.

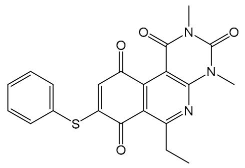

001
Molecular Weight: 407.4440

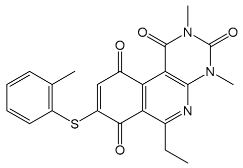

002
Molecular Weight: 421.4710

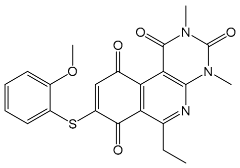

003
Molecular Weight: 437.4700

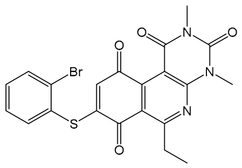

004
Molecular Weight: 486.3400

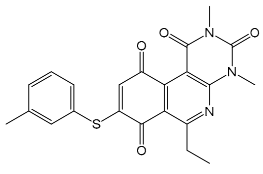

005
Molecular Weight: 421.4710

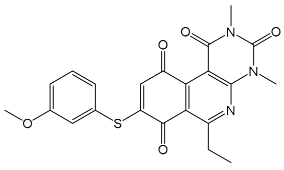

006
Molecular Weight: 437.4700

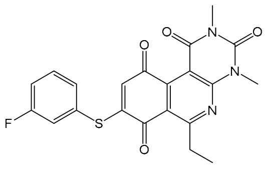

007
Molecular Weight: 425.4344

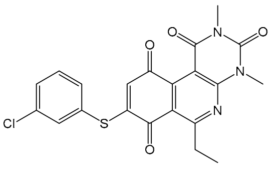

008
Molecular Weight: 441.8860

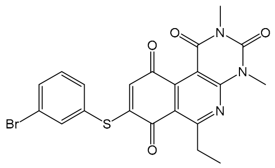

009
Molecular Weight: 486.3400

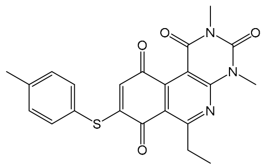

010
Molecular Weight: 421.4710

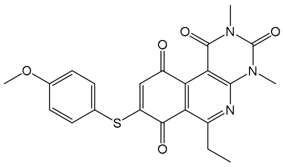

011
Molecular Weight: 437.4700

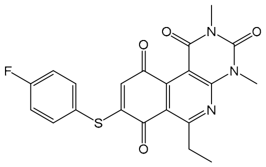

012
Molecular Weight: 425.4344

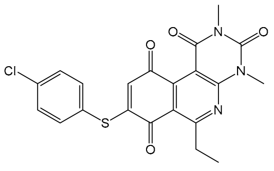

013
Molecular Weight: 441.8860

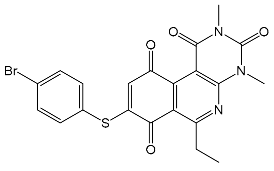

014
Molecular Weight: 486.3400

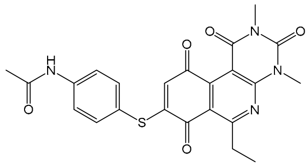

015
Molecular Weight: 464.4960

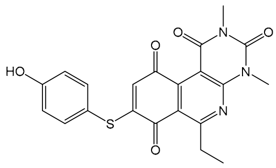

016
Molecular Weight: 423.4430

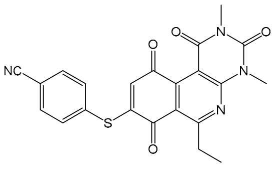

017
Molecular Weight: 432.4540

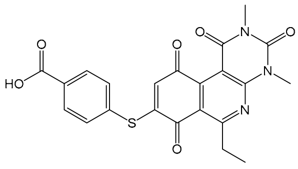

018
Molecular Weight: 451.4530

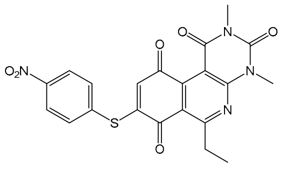

019
Molecular Weight: 452.4410

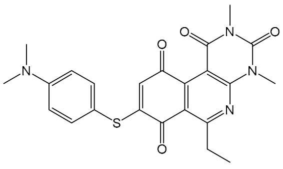

020
Molecular Weight: 450.5130

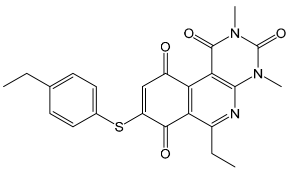

021

Molecular Weight: 435.4980

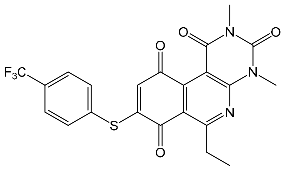

022

Molecular Weight: 475.4422

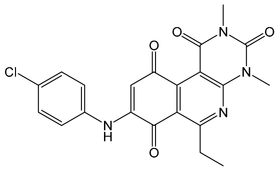

023

Molecular Weight: 424.8410

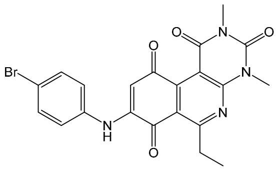

024

Molecular Weight: 469.2950

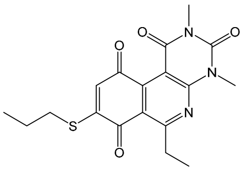

025

Molecular Weight: 373.4270

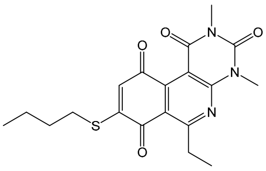

026

Molecular Weight: 387.4540

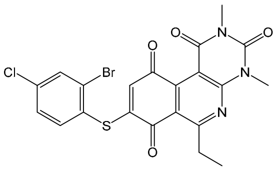

027

Molecular Weight: 520.7820

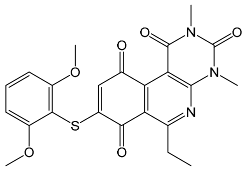

028

Molecular Weight: 467.4960

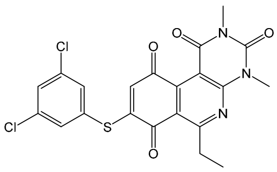

029

Molecular Weight: 476.3280

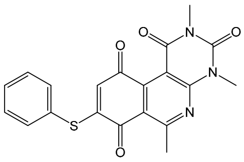

030

Molecular Weight: 393.4170

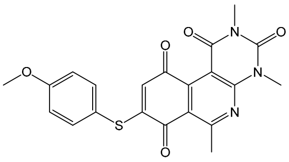

031

Molecular Weight: 423.4430

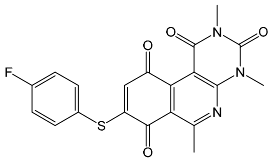

032

Molecular Weight: 411.4074

Figure S2. The chemical structure of the new designed and synthesized compounds.

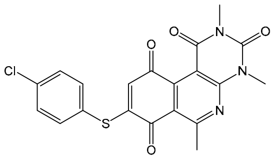

033
Molecular Weight: 427.8590

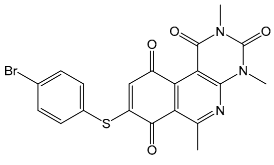

034
Molecular Weight: 472.3130

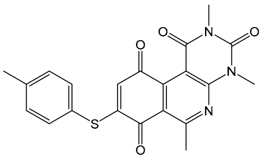

035
Molecular Weight: 407.4440

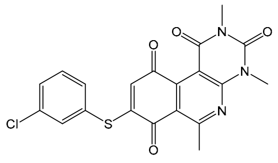

036
Molecular Weight: 427.8590

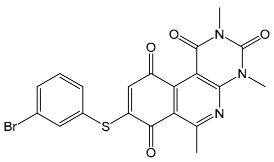

037
Molecular Weight: 472.3130

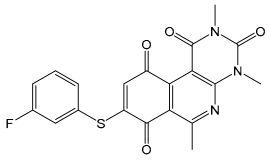

038
Molecular Weight: 411.4074

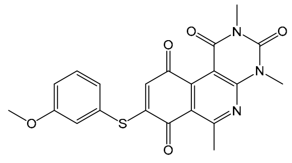

039
Molecular Weight: 423.4430

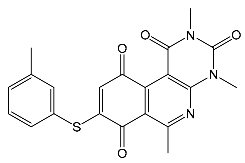

040
Molecular Weight: 407.4440

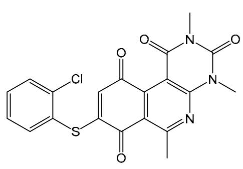

041
Molecular Weight: 427.8590

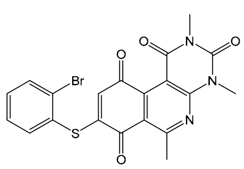

042
Molecular Weight: 472.3130

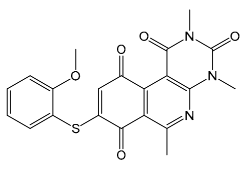

043
Molecular Weight: 423.4430

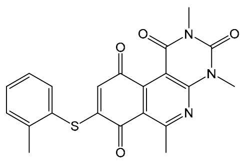

044
Molecular Weight: 407.4440

Figure S3. ^{1}H-NMR compound 33.

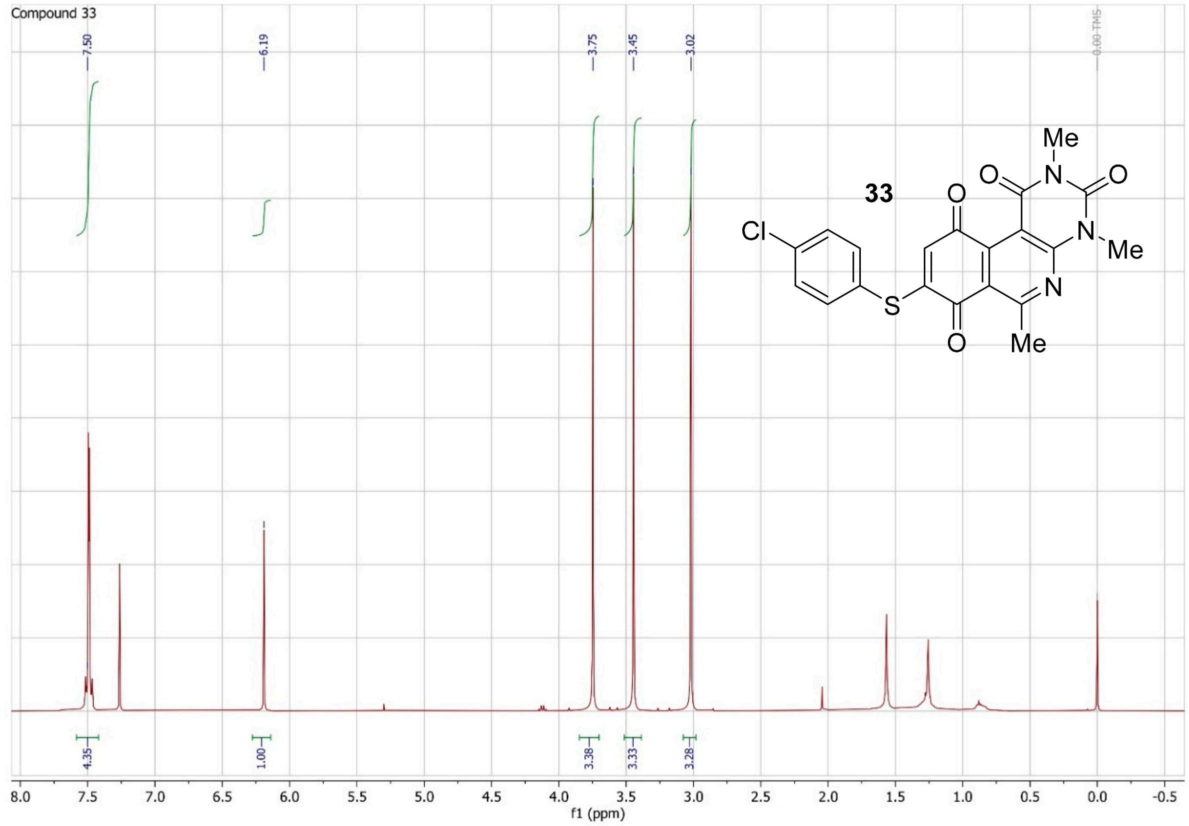

Figure S4. ^{1}H-NMR compound 34.

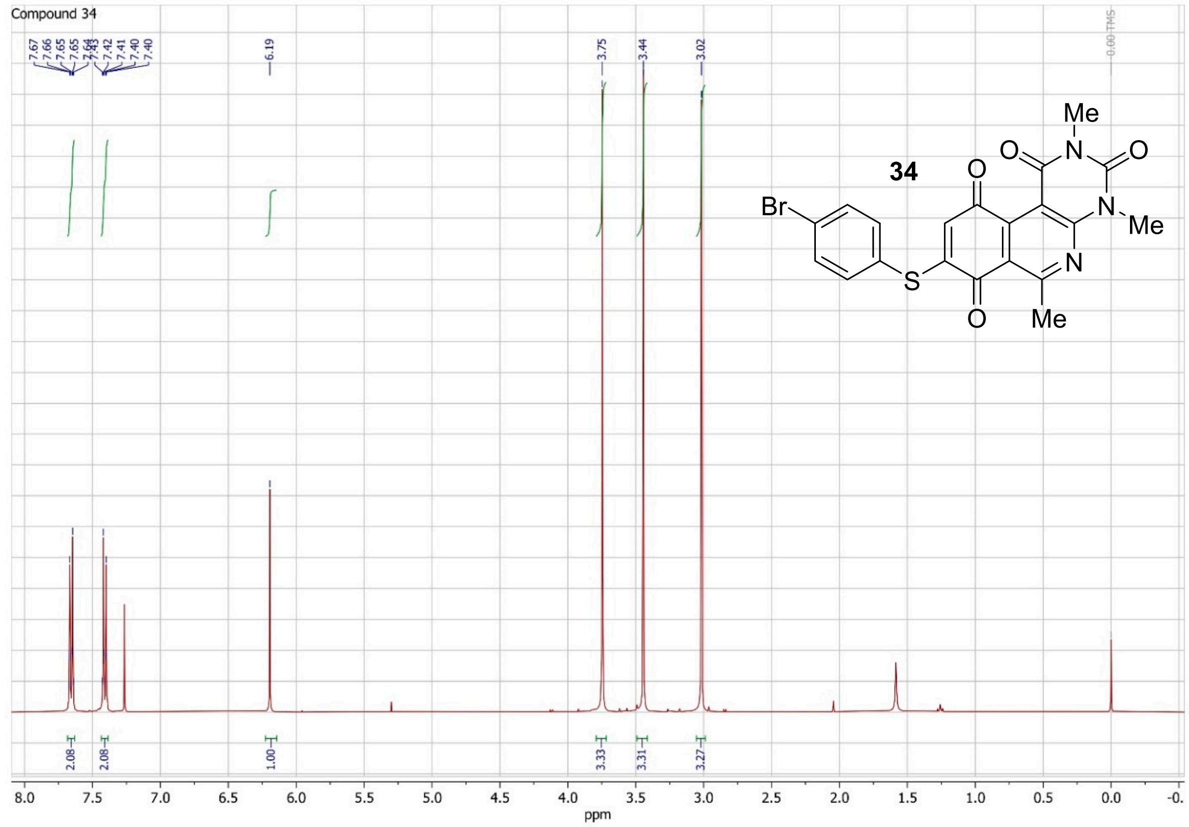

Figure S5. ^{1}H-NMR compound 35.

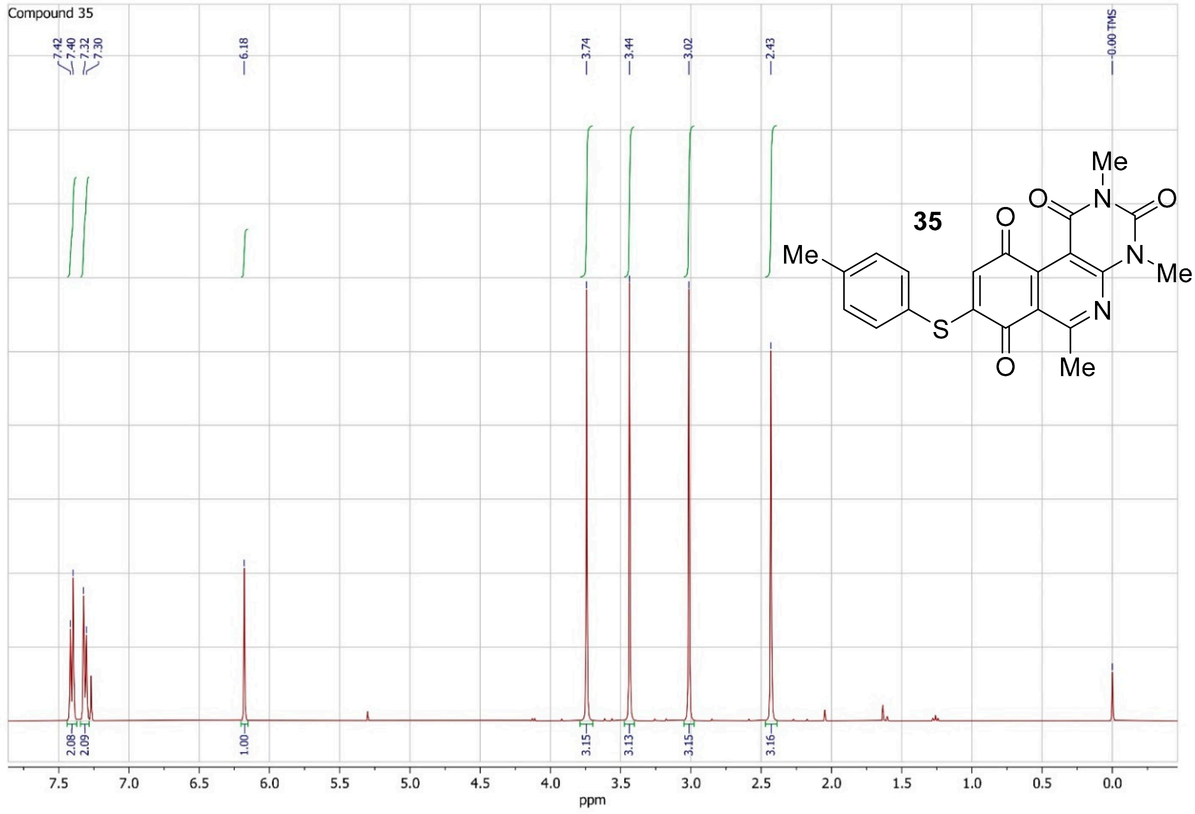

Figure S6. ^{1}H-NMR compound 36.

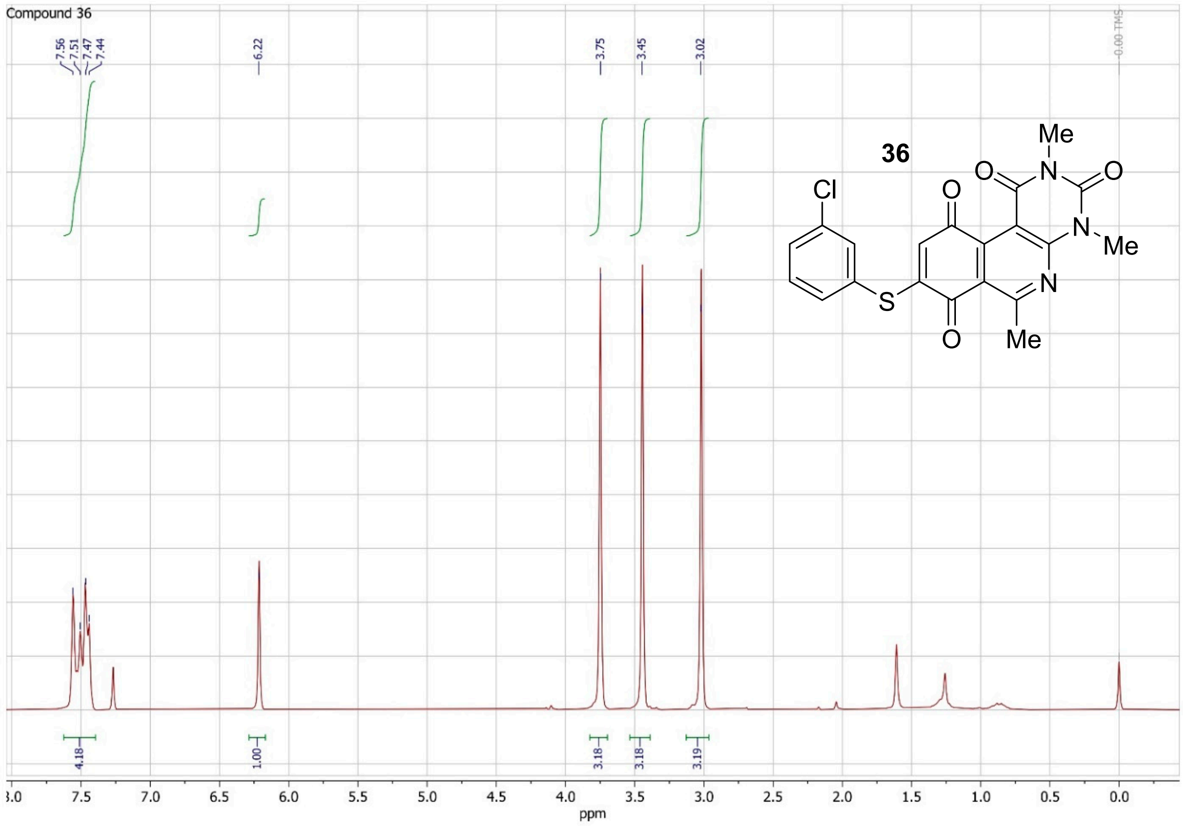

Figure S7. ^{1}H-NMR compound 37.

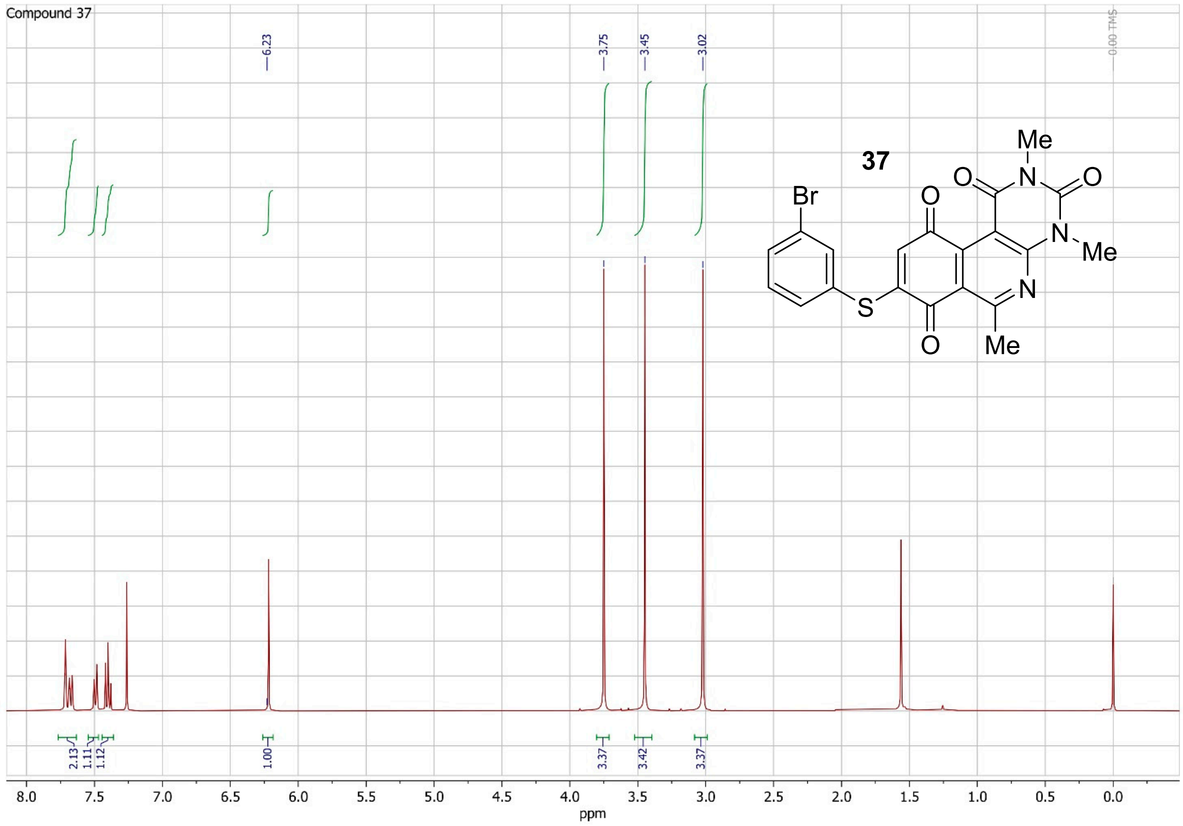

Figure S8. ^{1}H-NMR compound 38.

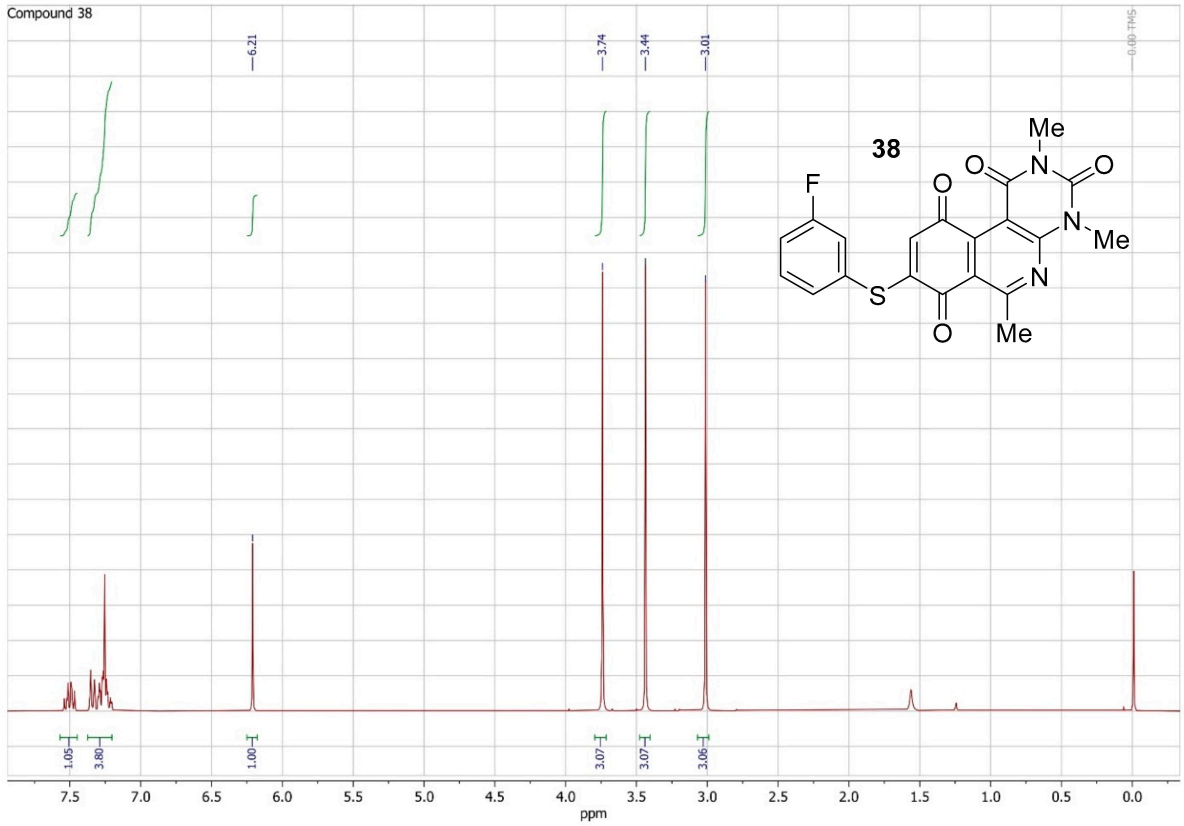

Figure S9. ^{1}H-NMR compound 39.

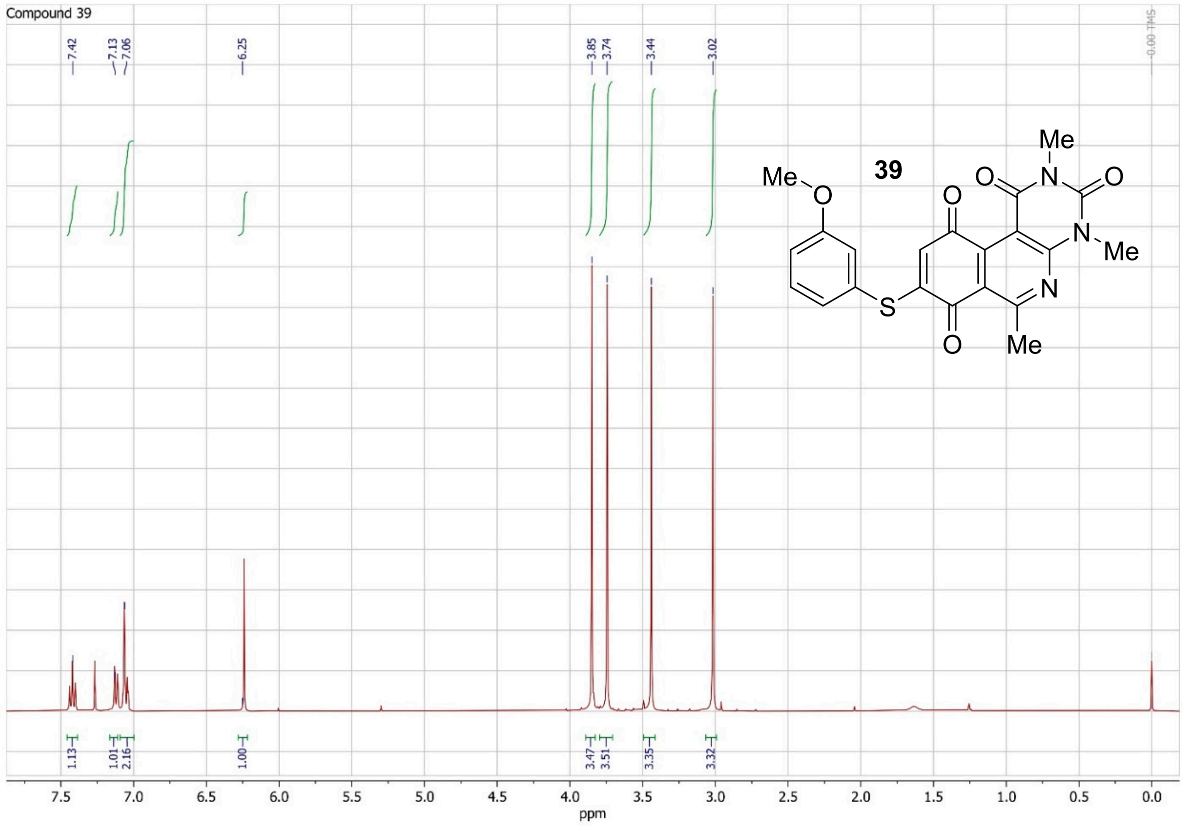

Figure S10. ^{1}H-NMR compound 40.

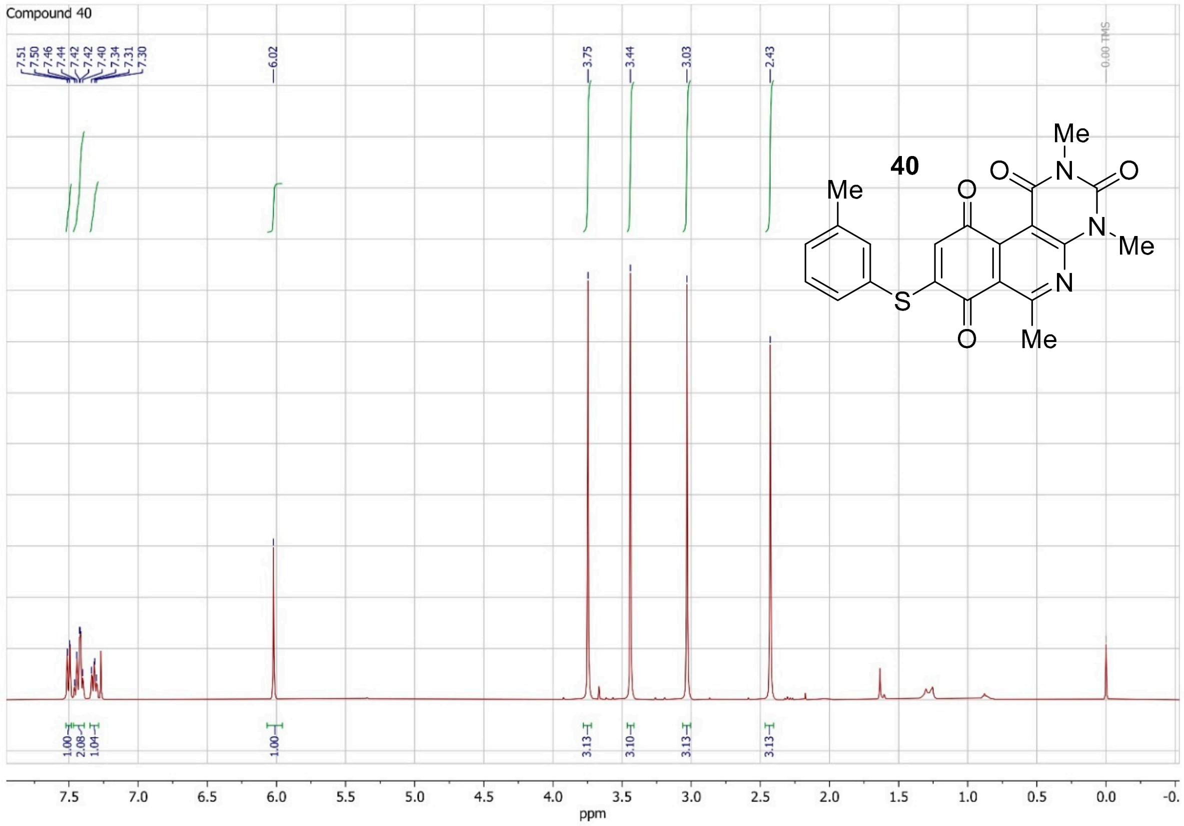

Figure S11. ^{1}H-NMR compound 41.

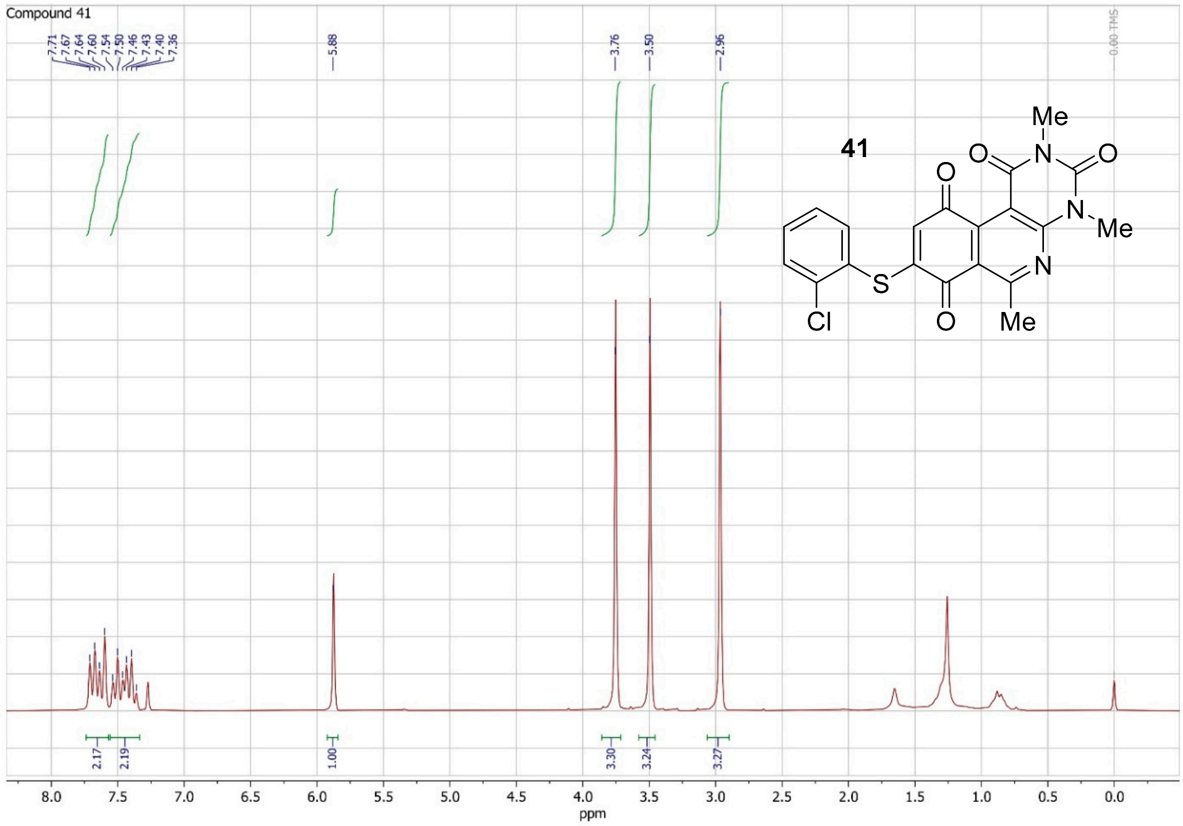

Figure S12. ^{1}H-NMR compound 42.

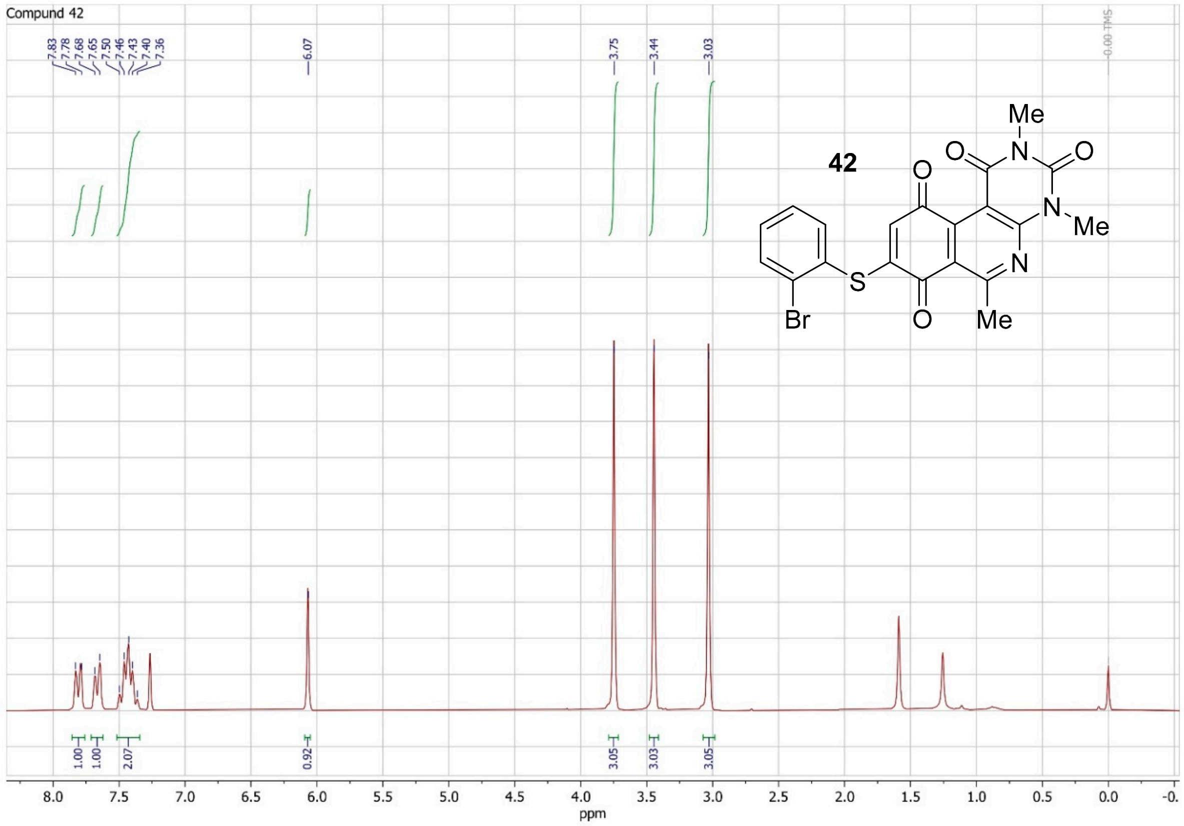

Figure S13. ^{1}H-NMR compound 43.

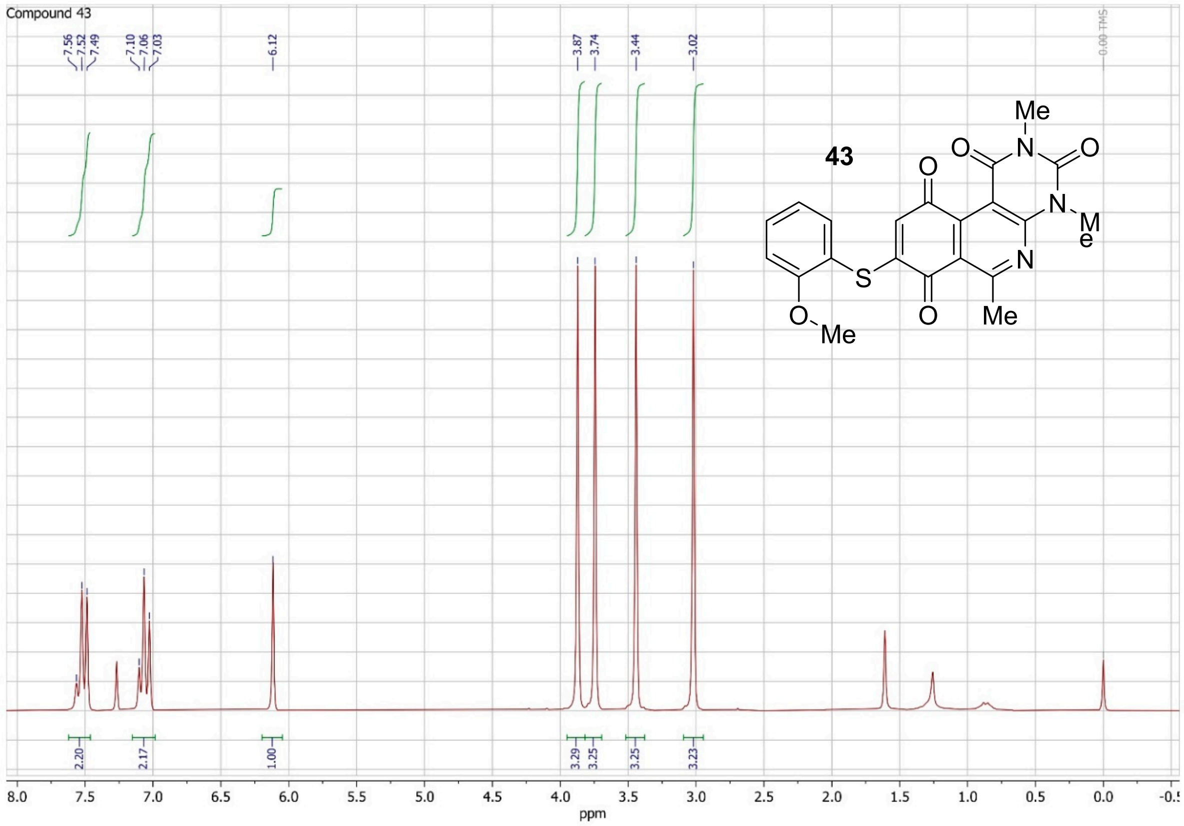

Figure S14. ^{1}H-NMR compound 44.

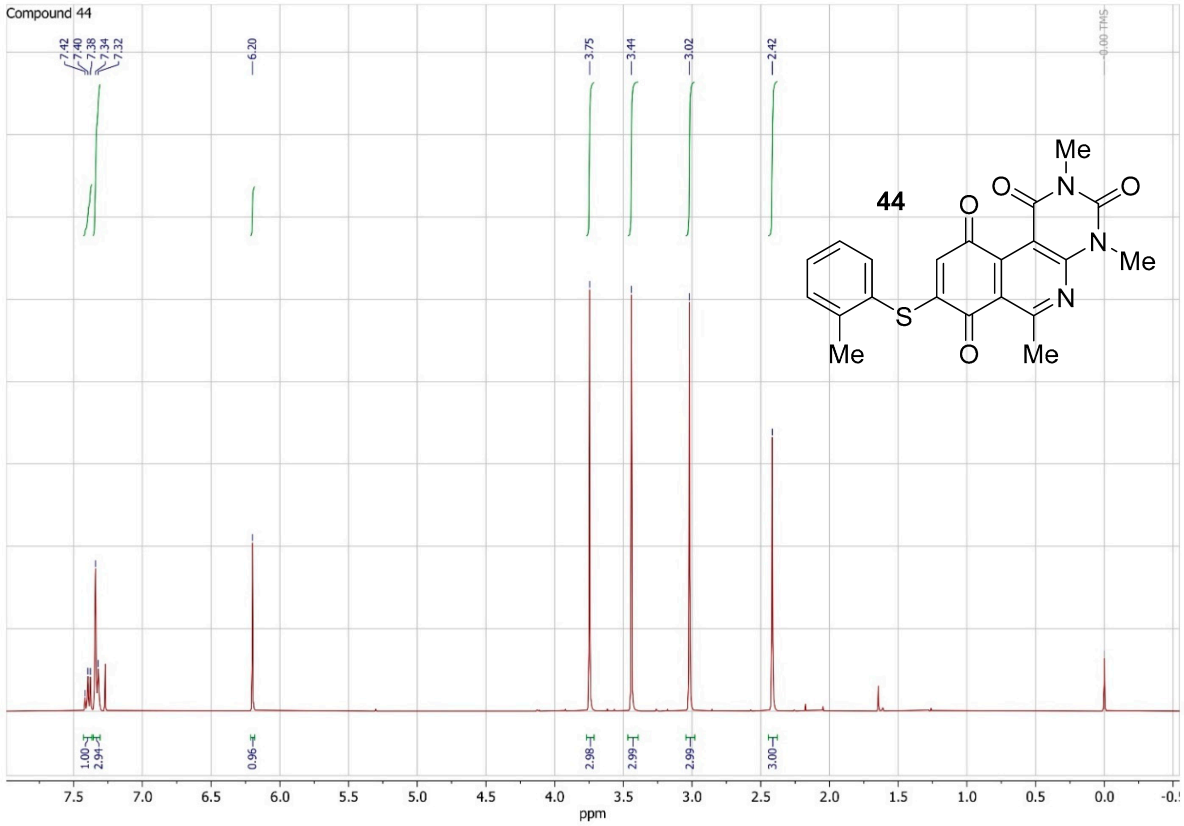

Figure S15. ^{1}H-NMR compound 45.

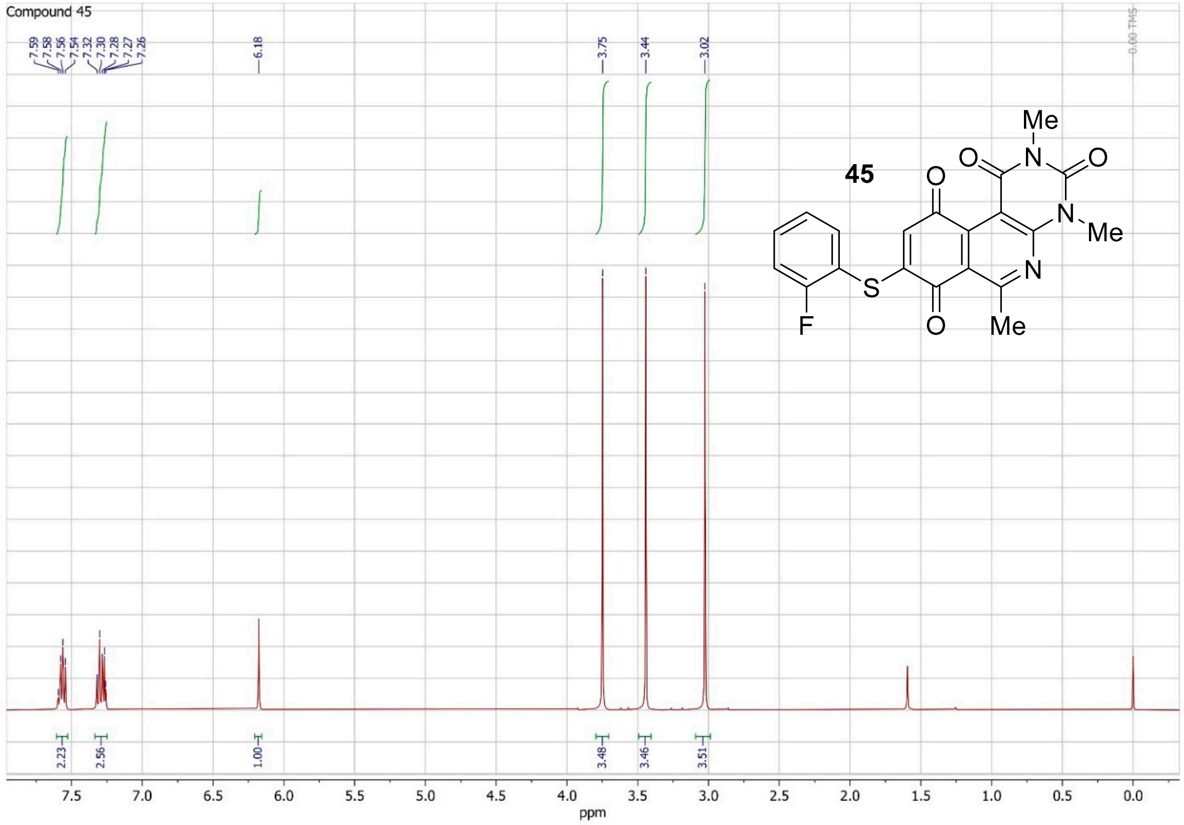

Table S1. The sequential search of the best CoMFA and CoMSIA models.

Model	q^2	N	SEP	SEE	r^2	F	Field Contributions				
							S	E	H	D	A
CoMFA-S	0.66	5	0.286	0.123	0.938	48.017	1				
CoMFA-E	0.541	2	0.305	0.212	0.779	33.54		1			
CoMFA-SE	0.619	3	0.286	0.14	0.909	59.703	0.659	0.341			
CoMSIA-S	0.591	5	0.314	0.219	0.801	12.857	1				
CoMSIA-E	0.482	2	0.324	0.217	0.769	31.585		1			
CoMSIA-H	0.237	1	0.383	0.31	0.502	20.169			1		
CoMSIA-D	0.416	3	0.354	0.303	0.572	8.03				1	
CoMSIA-A	0.275	5	0.275	0.303	0.618	5.17					1
CoMSIA-SE	0.468	2	0.329	0.226	0.748	28.158	0.418	0.582			
CoMSIA-SEH	0.526	13	0.457	0.021	0.999	651.809	0.132	0.34	0.528		
CoMSIA-SEHD	0.551	3	0.31	0.23	0.754	18.364	0.065	0.136	0.251	0.549	
CoMSIA-SEHA	0.534	14	0.506	0.025	0.999	431.371	0.113	0.252	0.518		0.117
CoMSIA-SED	0.515	4	0.331	0.227	0.773	14.492	0.188	0.302		0.41	
CoMSIA-SEA	0.596	5	0.312	0.159	0.895	27.276	0.269	0.504			0.227
CoMSIA-SEDA	0.52	5	0.34	0.223	0.794	12.332	0.175	0.353		0.275	0.197
CoMSIA-SH	0.393	2	0.351	0.243	0.71	23.247	0.271		0.729		
CoMSIA-SD	0.56	7	0.348	0.227	0.813	8.716	0.653			0.347	
CoMSIA-SA	0.397	5	0.381	0.218	0.802	12.956	0.55				0.45
CoMSIA-SHD	0.512	3	0.323	0.254	0.698	13.84	0.077		0.273	0.65	
CoMSIA-SHA	0.467	14	0.542	0.061	0.993	73.68	0.217		0.556		0.228
CoMSIA-SDA	0.494	8	0.387	0.227	0.827	0.827	0.576			0.258	0.166
CoMSIA-SHDA	0.507	15	0.562	0.066	0.993	58.439	0.203		0.532	0.131	0.134
CoMSIA-EH	0.505	15	0.564	0.021	0.999	596.284		0.4	0.6		
CoMSIA-ED	0.499	5	0.347	0.224	0.791	12.139		0.58		0.42	
CoMSIA-EA	0.574	4	0.311	0.187	0.846	23.398		0.688			0.312
CoMSIA-EHD	0.535	3	0.315	0.239	0.733	16.434		0.148	0.262	0.591	
CoMSIA-EHA	0.526	16	0.604	0.024	0.999	412.994		0.288	0.576		0.136
CoMSIA-EDA	0.502	5	0.346	0.23	0.781	11.386		0.465		0.286	0.249
CoMSIA-EHDA	0.568	5	0.568	0.136	0.924	38.632		0.224	0.37	0.241	0.164
CoMSIA-HD	0.477	3	0.335	0.268	0.664	11.862			0.273	0.727	
CoMSIA-HA	0.443	14	0.443	0.075	0.99	48.305			0.764		0.236
CoMSIA-HDA	0.454	2	0.333	0.279	0.615	15.186			0.117	0.749	0.133
CoMSIA-DA	0.439	3	0.347	0.298	0.586	8.502				0.842	0.158
CoMSIA-ALL	0.571	5	0.321	0.137	0.922	37.606	0.088	0.192	0.341	0.239	0.139
